# Worm Gear Drives with Improved Kinematic Accuracy

**DOI:** 10.3390/ma14247825

**Published:** 2021-12-17

**Authors:** Wojciech Kacalak, Maciej Majewski, Zbigniew Budniak, Jacek Ponomarenkow

**Affiliations:** 1 Faculty of Mechanical Engineering, Koszalin University of Technology, Racławicka 15-17, 75-620 Koszalin, Poland; zbigniew.budniak@tu.koszalin.pl (Z.B.); jacek.ponomarenkow@s.tu.koszalin.pl (J.P.); 2 Faculty of Mechanical Engineering and Ship Technology, Gdańsk University of Technology, Narutowicza 11/12, 80-233 Gdansk, Poland; maciej.majewski@pg.edu.pl

**Keywords:** worm gear drives, backlash elimination, worm wheels, worms, kinematic accuracy, mechanical design

## Abstract

This paper presents the fundamentals of the design and applications of new worm gear drive solutions, which enable the minimisation of backlash and are characterised by higher kinematic accuracy. Different types of worm surfaces are briefly outlined. Technological problems concerning the principles of achieving a high degree of precision in machining are also described. Special attention is paid to the shaping of conical helical surfaces. Increasing the manufacturing precision of drive components allows one to achieve both lower backlash values and lower levels of its dispersion. However, this does not ensure that backlash can be eliminated, with its value being kept low during longer periods of operation. This is important in positioning systems and during recurrent operations. Various design solutions for drives in which it is possible to reduce backlash are presented. Results of experiments of a worm gear drive with a worm axially adaptive only locally, in its central section, are presented. In this solution, it is possible to reduce backlash by introducing adjustment settings without disassembling the drive. An important scientific problem concerned defining the principles of achieving a compromise between the effectiveness of reducing backlash and the required load capacity of the drive. In this paper it has been shown that in worm gear drives with a locally axially adaptive worm, as well as with a worm wheel with a deformable rim, it is possible to achieve significant reduction of backlash. In high precision drives—for example, those with an average backlash value of <15 micrometers—this can enable more than a two-fold reduction of the average backlash value and more than a three-fold decrease of the standard deviation of local backlash values.

## 1. Introduction

Worm gear drives are widely used mostly in devices that require high precision and uniformity of motion transmission, especially in positioning the mechanisms of machine tools and measuring systems. Even the most precisely manufactured conventional drives, including planetary gears, which are very expensive, do not ensure backlash-free operation of the kinematic chain as they always possess some backlash, resulting from size deviations and shape inaccuracies of the drive components. Moreover, this backlash increases with progressive wear of the mating surfaces. It therefore follows that one way to ensure long-term backlash-free operation of kinematic systems is to use drives with adjustable backlash settings.

The aim of our experiments was to develop the basis for the design and application of new worm gear drive solutions that could provide a large range of good adjustment results, especially for minimising backlash, along with ensuring a high degree of kinematic accuracy. It was assumed that minimisation of backlash in the drive would be adaptive as a result of local deformations, and that the adjustment setting would be introduced without having to disassemble the drive.

Helical surfaces are widely used as the aggregation of rectilinear and circumferential displacements often occurs in technical systems. Helical surfaces most frequently occur in the form of thread coils, gears, worms, worm wheels, drill groove surfaces, pumping systems, and other working parts in mechanical systems.

Ball screw drives are used in drive and positioning systems. Worm gear drives, however, are used in many kinematic systems, including indexing mechanisms in machine tools, indexing heads, rotary tables, in car steering gears, worm reducers in positioning systems, and ship propulsion control systems. Processes in the improvement of worm gear drive design and technology have contributed to the reduction of backlash [1], an increase in durability [2], design optimisation [3], the improvement of contact conditions of mating surfaces [4,5], design modification [6,7], the application of new grinding wheels for grinding worm screw surfaces [8,9], advanced analyses and methods [10,11], an increase in the precision of technological processes, as well as an improvement in the control methods of these processes [12]. This ensures that the quality of kinematic systems with a high degree of stiffness and load capacity, along with the range of their applications, are systematically increasing [5].

A new range of worm gear drive applications has been described in the study [13]. The authors presented a dynamic model of a worm gear drive, in which self-locking can be reduced by means of vibrations and the motion transmission between the worms and worm wheels can be changed. In mechanisms in which there is a displacement of the worm axis, thereby causing deviation of the axis position angle, the efficiency of the drive decreases [14]. The methodology for creating surface models in the meshing zone, as described in [15], can also be used for different types of meshing.

In the search for new innovative solutions, new design methods with the implementation of creative problem-solving techniques [16,17] have been helpful, as well as design anti-patterns using methods based on artificial intelligence [17,18].

The processes of helical surface formation are accompanied by high requirements concerning pitch accuracy, as the accuracy of positioning displaced components depends on this and the accuracy of other elements of a kinematic node [19]. On the other hand, the load capacity and durability of the drive depends on the precision of the surface outline of the mating components of the drive, as well as the stereometric properties of the surface and the physical properties of the surface layer [1].

The number of different types of helical surfaces is not limited. Helical surfaces can constitute surfaces formed by helical lines located on a cylinder, cone or any other surface. Even among the surfaces formed by lines on a cylinder, one can distinguish between rectilinear and non-rectilinear surfaces, i.e., those for which it is possible to determine a line which is straight in a certain plane and those which do not possess a straight line in any cross-section. For practical reasons, however, standardisation is implemented while standardised surface types are established. The distinguishing factor is the shape of the outline in a certain plane cross-section. In this way, only rectilinear surfaces are distinguished.

Nowadays, the most commonly used surfaces are, among others, helical surfaces for which no surface outline is defined, although the nominal outline of the tool is defined for machining them. These surfaces are non-rectilinear with the definition of the outline being as follows:

- conical worm *K*, whose nominal cross-section is a normal cross-section and whose nominal outline of the rotary tool is a straight line,
- toroidal worm KR, whose nominal cross-section is a normal cross-section and the nominal outline of the rotating tool is a circular arc.

The shape of the tool is therefore the distinguishing feature of non-rectilinear surfaces. However, it must be taken into account that in order to determine the shape fully, the geometrical parameters of the tool must also be specified.

Helical surfaces of workpieces with a hardness of less than 36 HRC can be machined using turning or milling operations with special tools and small cross-sections of the cut layers. Precision helical surfaces of components with a hardness above 36 HRC are usually shaped by grinding.

The grinding of helical surfaces is a complex process in terms of tool selection and shaping, as well as the accuracy of tool positioning and the selection of grinding parameters to ensure the required performance and good properties of the surface layer. Helical surfaces can be ground using a variety of tools [20,21]—disc grinding wheels, finger grinding wheels, ring grinding wheels or cup wheels. Although grinding with internal inserts permits high grinding speeds, the shaping of such grinding wheels is not easy. In addition, there are numerous restrictions on geometrical parameter relationships.

The use of finger grinding wheels does not ensure high grinding efficiency as the stiffness of these grinding wheels is too low [21]. Finger grinding wheels are characterised by a small active surface area, possess low shape retention and require frequent renewal of the surface. In some grinding operations on helical surfaces—for example in the process of machining worm cutter blades—finger grinding wheels can be used as they allow one to obtain the required outline of the ground surface and the shape of the cutter teeth’s contact surface. Grinding wheels and ring-shaped wheels are of limited use as it is not possible to grind both sides of a cut-out simultaneously with them.

The most frequently used wheels are ring grinding wheels, which possess a high degree of stiffness, have a large active surface area and can be used to grind both sides of an internal cut-out simultaneously, providing a high grinding capacity. However, the use of grinding wheels requires the application of complex systems for shaping the wheel’s active surface, such that a outline corresponding to a defined type of helical surface is obtained. Only in the case of the grinding of non-rectilinear helical surfaces is a rectilinear tool outline easy to achieve.

In industrial settings, the geometrical analysis of the helical surface grinding process is often too cursory. The mathematical relationships describing the geometrical characteristics of axial or normal outlines are complex, especially in the case of conical and toroidal surfaces. Deviations from the correct nominal wheel outline for grinding a particular helical surface are sometimes allowed. These may be allowed when an error in the outline of the helical surface caused by a deviation from the nominal outline of the grinding wheel is small in relation to the permissible outline deviation, with the curvature of the outline being slightly increased.

Deviations from the nominal outline of the grinding wheel surface, permissible due to the operational characteristics, are usually caused by a need to simplify the technological process and to increase productivity or reduce manufacturing costs. Occasionally, deviations may be due to the impossibility of using technological devices that could produce a helical surface with the nominal outline.

**Figure 1:**
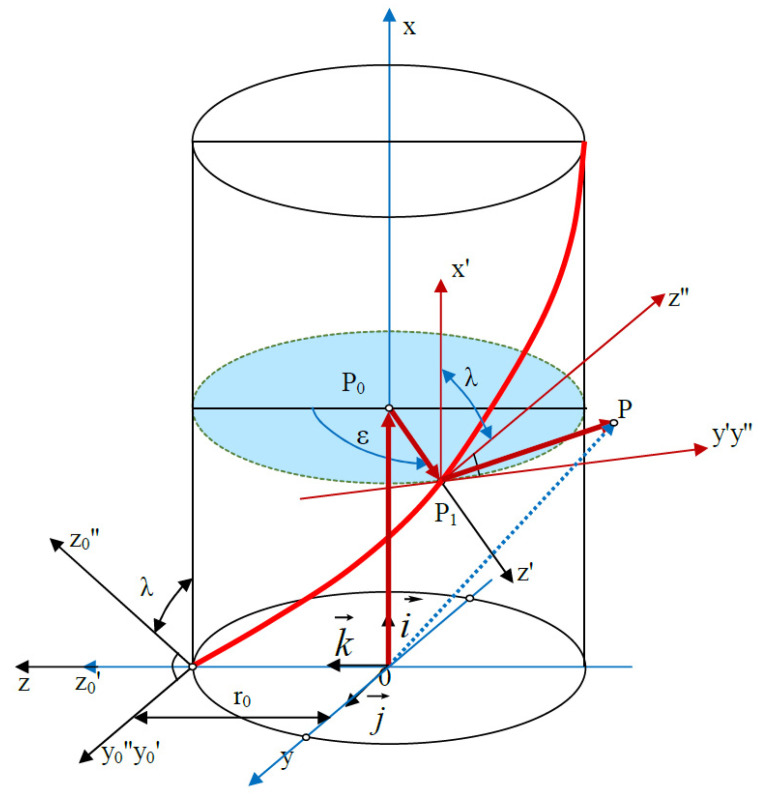
Vector of helical surface.

The general equation of helical surface (1) possesses special cases which are obtained by taking the following into account (Figure 1):

- the position of the plane in which the outline of the helical surface is defined, that is the value of the angle λ and the distance $r_{0}$ when λ=0,
- the forms of the outline functions ψ(u) and ϕ(u) in this plane, described by implicit correlations for indeterminate surfaces.

(1) $$\begin{array}{cc} \vec{OP} & =\left[\varphi \left(u\right)\cos\lambda +P\frac{\varepsilon }{2\pi }\right]\vec{i}+\left[\varphi \left(u\right)\sin\lambda \sin\varepsilon +\psi \left(u\right)\cos\varepsilon +r_{0}\sin\varepsilon \right]\vec{j}+\\ \; & +\left[\varphi \left(u\right)\sin\lambda \cos\varepsilon -\psi \left(u\right)\sin\varepsilon +r_{0}\cos\varepsilon \right]\vec{k} \end{array}$$

**Figure 2:**
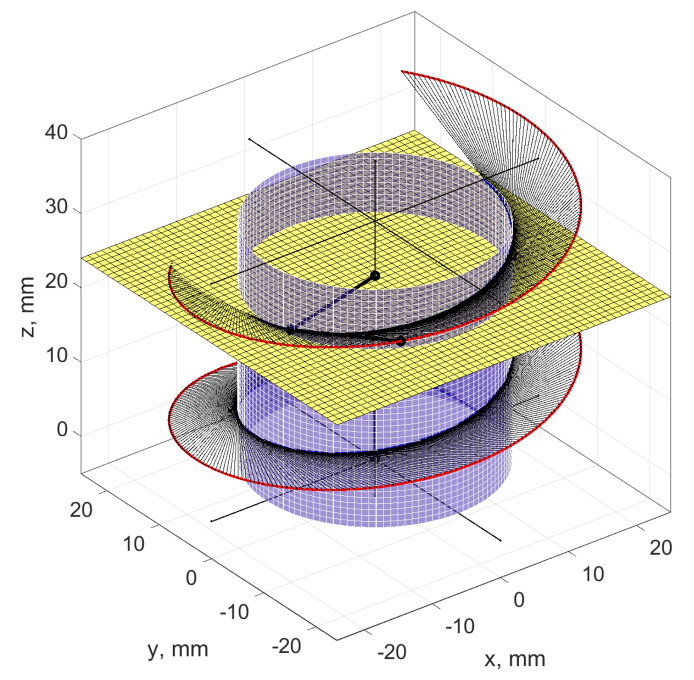
Involute helical surface.

One example of a rectilinear helical surface is an involute helical surface whose outline is involute in a frontal section (Figure 2).

**Figure 3:**
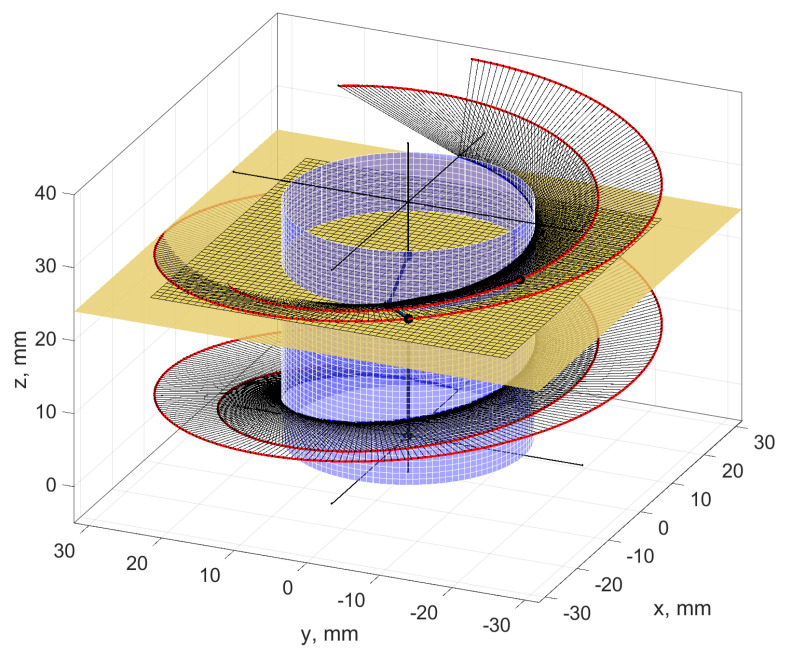
Convolute helical surface.

Another type of simple helical surface is the convolute helical surface. A convolute (elongated involute) is an outline in the frontal cross-section, while the surface has a rectilinear outline in the cross-section perpendicular to the helical line on the central cylinder (Figure 3).

**Figure 4:**
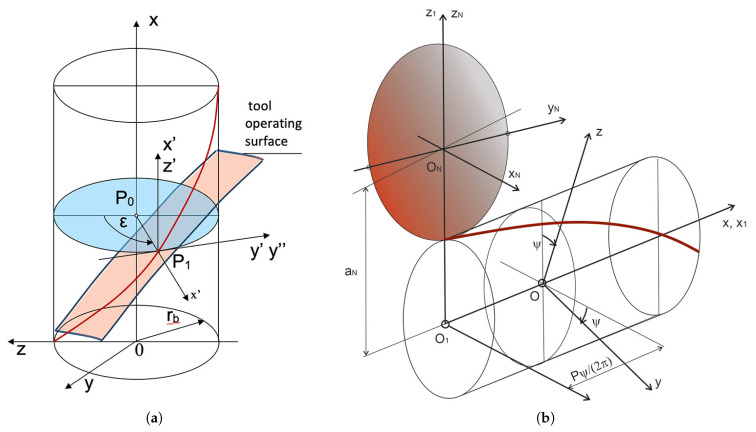
Coordinate system for the analysis of the shaping process of a non-rectilinear helical surface. (**a**) Non-rectilinear helical surface as a general case of enveloping the tool surface in helical motion. (**b**) Helical surface created by shaping with a tool with a defined axial outline.

Non-rectilinear helical surfaces are formed as the enveloping of the tool surface in helical motion (Figure 4a,b). If the tool outline is rectilinear (trapezoidal) and the active surface of the tool is a double cone, a helical cone surface is formed.

**Figure 5:**
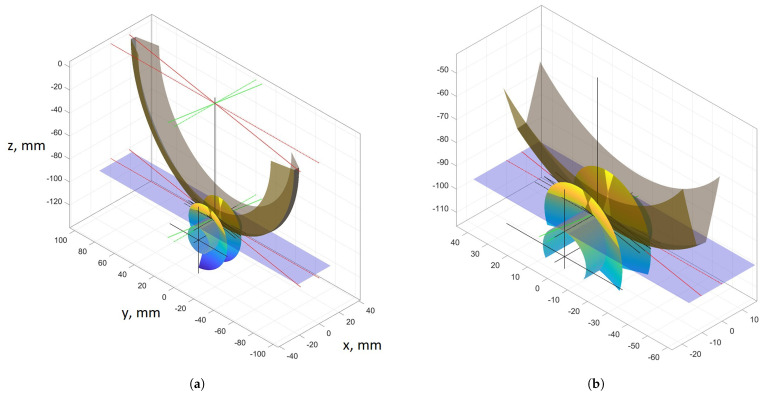
Shape of the grinding zone of a conical helical surface. (**a**) Image view of the zone. (**b**) Enlarged image of the zone.

The axial outline of such surfaces is convex and is not rectilinear in any other cross-section. When the helical surface is ground with a conical grinding wheel and, in an earlier preliminary operation, has obtained a rectilinear outline in the axial cross-section as a result of turning, the shape of the grinding zones will be variable along the height of the coil (Figure 5).

## 2. Shape Analysis of Conical Surfaces

Worm gear drives with a cone-shaped helical surface are characterised by their ease of manufacture due to the simple shapes of rotary tools. The tool has a rectilinear axial outline and can machine both sides of the cut-out simultaneously.

**Figure 6:**
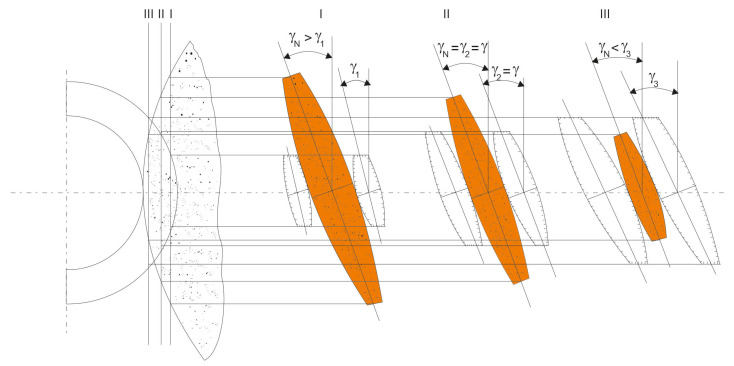
Causes of deviation from straightness of the axial outline of the helical surface; $\gamma_{1}$, $\gamma_{2}$, $\gamma_{3}$—inclination angles of tangents to the helical surface outline on the diameter (in section I, II, III); $\gamma_{N}$—inclination angle of the grinding wheel plane of symmetry.

The axial outline of the conical helical surface is convex. This is due to the fact that the plane of symmetry of the rotary tool is parallel to the tangent to the helical surface only on one specific rotary tool (Figure 6), e.g., the indexing roller (no parallelism in other positions).

The authors have developed an application for the calculation and analysis of the axial contour geometry of conical screw surfaces.

**Figure 7:**
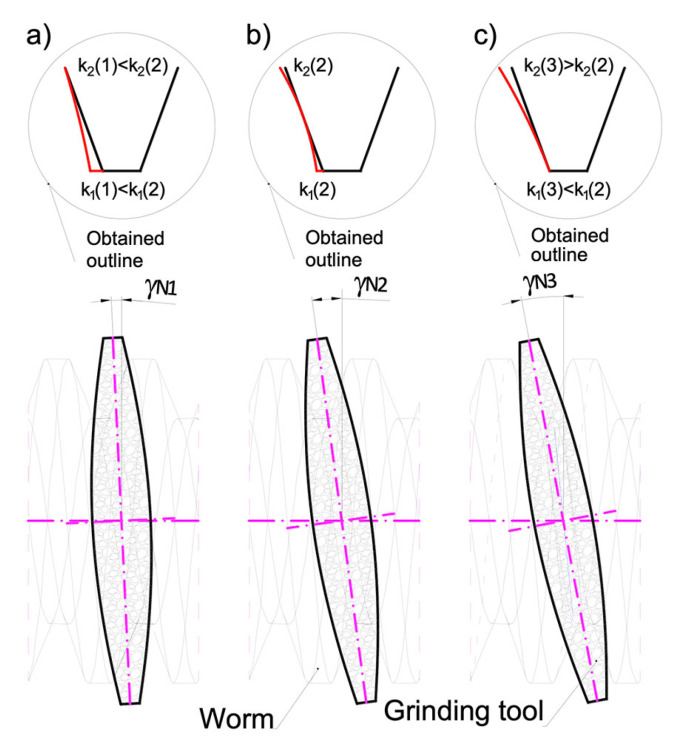
Influence of the grinding wheel axis inclination angle on the shape of the obtained axial outline of conical helical surface and deviations from straightness $k_{1}$ and $k_{2}$.

The inclination angle of the grinding wheel axis (Figure 7) influences the shape of the obtained axial outline of the conical helical surface:

I inclination angle of the axle of the grinding wheel lower than the angle of elevation of the helical line on the indexing roller,
II inclination angle of the rotating grinding wheel axis equal to the helix angle on the indexing roller,
III inclination angle of the grinding wheel axis greater than the angle of helix inclination on the indexing roller.

The operating conditions of worm gear drives are strongly influenced by deviations in the outline of ground helical surfaces. It has been shown in study [21] that the most important causes of deviations of this outline can be reduced to three direct causes, namely:

- deviations of the position of the grinding wheel axis in relation to the workpiece axis,
- deviations of the inclination angle of the grinding wheel axis in relation to the workpiece axis,
- deviations of the outline of the grinding wheel.

The deviation of the inclination angle of the grinding wheel axis results mainly from the deviation of the setting of this angle, as well as the deviation of the position of the grinding wheel axis in relation to the helical surface axis. This positional deviation is caused by geometrical inaccuracy and deformations of the machine tool, along with deformations of the workpiece itself.

A deviation of the grinding wheel outline may be caused by a deviation of a grinding wheel after dressing caused by technological inaccuracy of dressing machine and its settings. Additionally, this may be caused by the adoption of an alternative outline. The deviation of the grinding wheel outline also results from the process of shape wear. An analysis of calculation results indicates that deviation of the grinding wheel outline caused by its wear, or the application of an alternative outline, has a decisive influence on the outline deviation value [21].

The relationships for determining the deviations from straightness $k_{1}$ and $k_{2}$ are complex (2) at the base of coil $k_{1}$, at the pitch diameter $k_{0}\left(k\right)$ and at the outer diameter $k_{2}$. The parameter assigned to the values of $k_{1}$, $k_{0}$, $k_{2}$ is $A_{m}\left(4\right)$, for which i={0,1,2} takes the values [0, −1, 1] corresponding to the points on the pitch diameter, the diameter of the bases and the outer diameter of the helical surface of the worm.

(2) $$\begin{array}{cc} k_{1} & =m\cdot \tan\alpha_{0}-[\left(u_{1}-u_{0}\right)\cdot \sin\alpha_{N}\cdot \cos\cdot \left(\arctan\frac{m\cdot z_{1}}{d}\right)+\\ \; & +\left(u_{1}\cdot \sin\xi_{1}-u_{0}\cdot \sin\xi_{0}\right)\cdot \cos\alpha_{N}\cdot \sin\cdot \left(\arctan\frac{m\cdot z_{1}}{d}\right)+\\ \; & +\left(\psi_{0}-\psi_{1}\right)\cdot \frac{m\cdot z_{1}}{2}],\\ k_{2} & =m\cdot \tan\alpha_{0}-[\left(u_{2}-u_{0}\right)\cdot \sin\alpha_{N}\cdot \cos\cdot \left(\arctan\frac{m\cdot z_{1}}{d}\right)+\\ \; & +\left(u_{2}\cdot \sin\xi_{2}-u_{0}\cdot \sin\xi_{0}\right)\cdot \cos\alpha_{N}\cdot \sin\cdot \left(\arctan\frac{m\cdot z_{1}}{d}\right)+\\ \; & +\left(\psi_{0}-\psi_{2}\right)\cdot \frac{m\cdot z_{1}}{2}], \end{array}$$

where: $z_{1}$—the number of turns of the helical surface, and the parameters $u_{i}$, $\xi_{i}$, $\Psi_{i}$—are determined from Equations (3)–(5):

(3) $$\begin{array}{cc} u_{i} & =\left[d_{N}\cdot \tan\alpha_{N}+\frac{\pi \cdot m}{4}\cdot \cos\left(\arctan\frac{m\cdot z_{1}}{d}\right)\right]\cdot \sin\alpha_{N}-\\ \; & -\left[\left(\frac{d_{N}}{2}+\frac{d}{2}\right)\cdot \sin\alpha_{N}+\frac{m\cdot z_{1}}{2}\right]\cdot \tan\xi_{i}-\\ \; & -\frac{\left(\frac{d_{N}}{2}+\frac{d}{2}-\frac{m\cdot z_{1}}{2}\right)\cdot \cos\alpha_{N}}{\cos\xi_{i}}, \end{array}$$

(4) $$\xi_{i}=\arccos\left(\frac{\left(\frac{d}{2}+A_{m}\cdot m\right)\cdot \Psi_{i}-\left(\frac{d_{N}}{2}+\frac{d}{2}\right)}{u_{i}\cdot \cos\alpha_{N}}\right),$$

(5) $$\begin{array}{cc} \Psi_{i} & =\arctan\{\frac{u_{i}\cdot \left[\cos\alpha_{N}\cdot \sin\xi_{i}\cdot \cos\left(\arctan\frac{m\cdot z_{1}}{d}\right)-\sin\alpha_{N}\cdot \sin\left(\arctan\frac{m\cdot z_{1}}{d}\right)\right]}{u_{i}\cdot \cos\alpha_{N}\cdot \cos\xi_{i}+\frac{d_{N}}{2}+\frac{d}{2}}+\\ \; & +\frac{\left[d_{N}\cdot \tan\alpha_{N}+\frac{\pi \cdot m}{4}\cdot \cos\left(\arctan\frac{m\cdot z_{1}}{d}\right)\cdot \sin\left(\arctan\frac{m\cdot z_{1}}{d}\right)\right]}{u_{i}\cdot \cos\alpha_{N}\cdot \cos\xi_{i}+\frac{d_{N}}{2}+\frac{d}{2}}\}, \end{array}$$

where: *m* is the module, $\alpha_{0}$ is the angle of the axial outline of the helical surface, $\alpha_{N}$ is the is the tool profile angle, $z_{1}$ is the number of turns of the worm, *d* is the pitch diameter, $d_{N}$ is the tool diameter corresponding to the pitch diameter of the helical surface.

**Figure 8:**
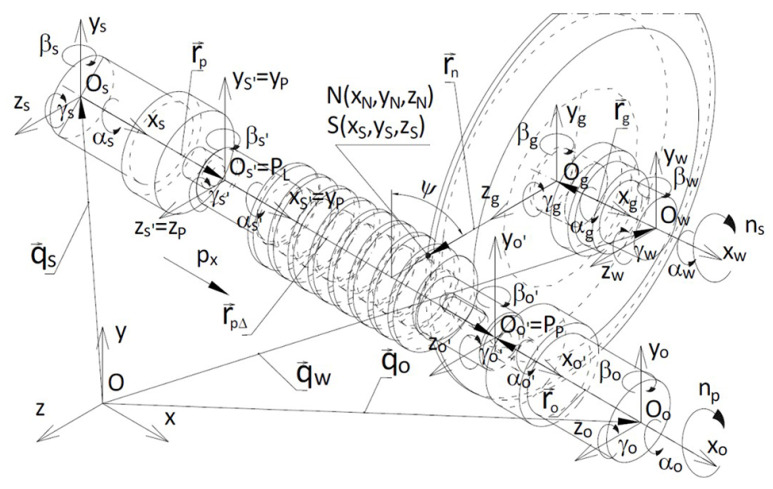
Shaping of the conical helical surface of the worm—kinematic system, coordinate systems of components for setting the machine tool, worm, grinding wheel, as well as vectors describing positions of components of the technological system.

In order to facilitate calculations, it is possible to develop a simulation model that enables numerical analyses of the CHOT (cutter-holder-object-tool) technological system in the process of grinding a conical helical surface, taking into account a numerous set of causes of inaccuracies in vector terms [22] (Figure 8).

This allows, among other things, for:

- modelling of the helical surface taking into consideration the influence of geometrical and kinematic inaccuracy of the machine tool, inaccuracy of the wheel outline after dressing, as well as inaccuracy of setting the machine;
- determination of multiple datasets and analysis of pitch deviations;
- analysis of deviations of the axial outline of the conical helical surface of the worm.

**Figure 9:**
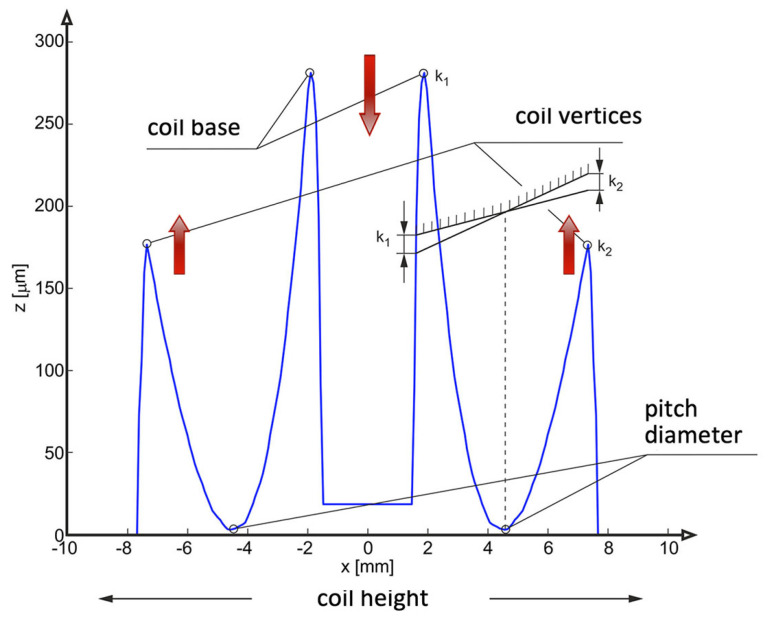
Projection on the x−z axial plane of the allowance values for grinding a helical surface with trapezoidal grinding wheel with rectilinear axial outline before the grinding operation.

The following conclusions concerning the shape of the conical surfaces result from the analyses carried out:

(1) By changing the inclination angle of the grinding wheel axis, it is possible to optimise the shape and properties of the meshing zone in the worm gear drive (Figure 9).
(2) By changing the inclination angle of the grinding wheel axis to one other than the inclination angle of the helical line on the central cylinder, it is possible to obtain such an angular position that the deviations from straightness of the nominal axial outline $k_{1}$ and $k_{2}$ will be equal to each other and smaller than $max(k_{1},k_{2})$.

Figure 9 shows the thickness of the grinding allowance on the left and right-hand side of the worm screw coil for the case when, before grinding, the worm was machined by turning and had a rectilinear axial outline. After grinding with a grinding wheel, the helical surface of the coil will be a conical surface. The value of $k_{1}$ indicates the deviation from straightness of the axial outline of the conically derived helical surface of the worm coil at the base of the coil (Figure 7).

## 3. Worm Gear Drives with Backlash Adjustment

Even the most precise conventional drives cannot provide backlash-free operation as there is backlash due to inaccuracies in the drive components, in their assembly or due to operational wear. The drives that can ensure backlash-free operation of a technical system are special worm gear drives with various shapes of helical surfaces [23,24,25], as well as a number of solutions that allow adjusting axial pitch by deforming meshing components or changing axial distance [26,27]. Design modifications may concern worms or worm wheels. Among drives with modified worms, the following can be distinguished:

- worm gear drives with a two-step worm,
- worm gear drives with a worm possessing a conical gearing surface,
- worm gear drives with a split worm,
- worm gear drives driven by two worms.

**Figure 10:**
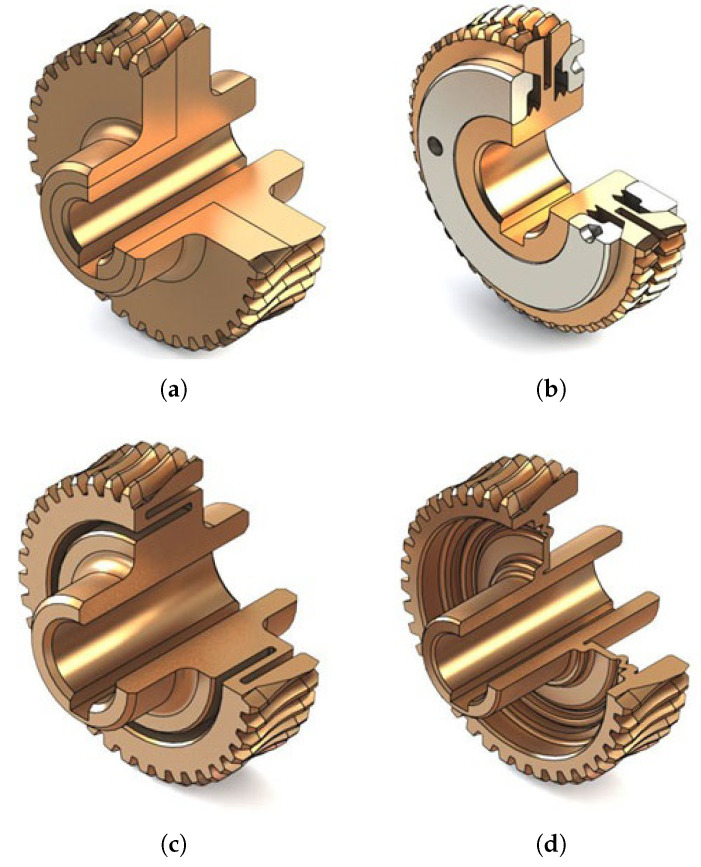
Examples of worm wheels with modified design. (**a**) Symmetrically split worm wheel. (**b**) Worm wheel with a rim split with circumferential cut-out. (**c**) Adaptive worm wheel with circumferential narrowing. (**d**) Adaptive worm wheel with connecting component.

**Figure 11:**
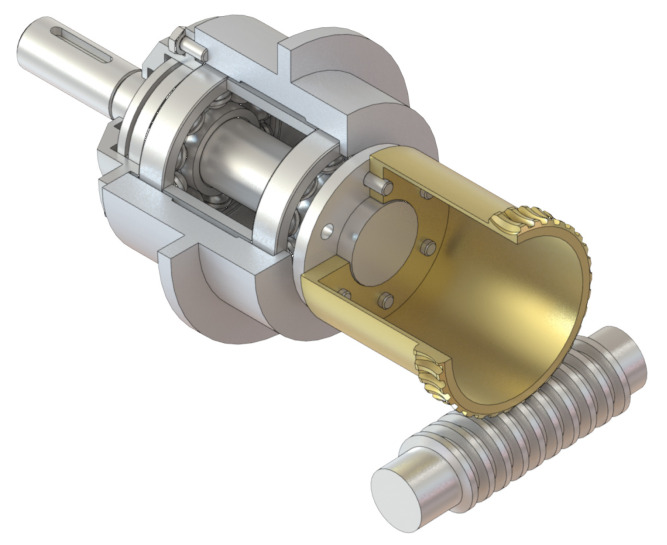
Worm gear drive with worm wheel rim positioned on a thin-walled sleeve.

**Figure 12:**
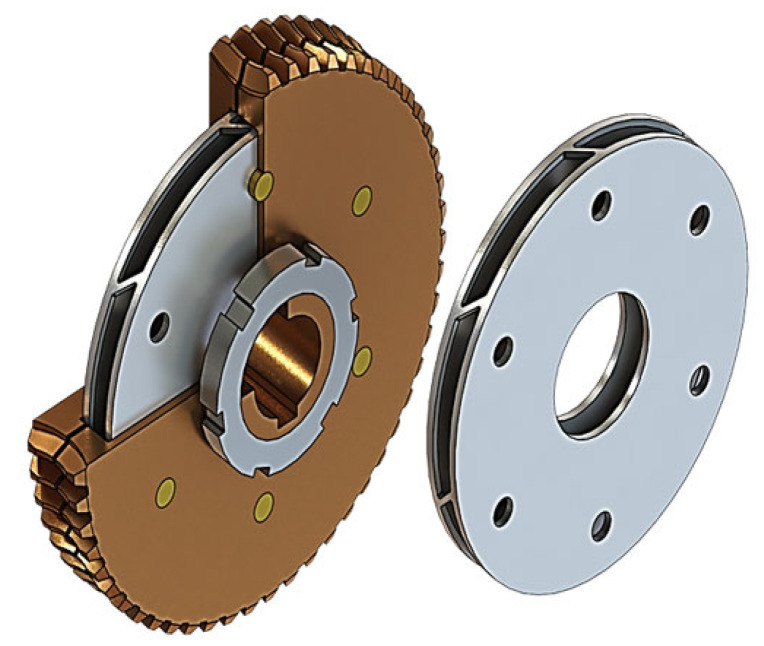
Split worm wheel with internally hollowed-out half and flexible disc converting a slight deformation towards the worm axis into rotational deformation around this axis.

Many new designs of zero-backlash worm gear drives are based on modified worm wheels, among which we can distinguish:

- symmetrically split worm wheels (Figure 10a),
- worm wheels with a rim split with a circumferential cut-out (Figure 10b),
- adaptive worm wheels with a circumferential narrowing (Figure 10c) or a connecting component (Figure 10d),
- Worm wheels situated on a radially deformable thin-walled sleeve (Figure 11),
- split worm wheel with an internally hollowed-out half and a flexible disc (Figure 12).

**Figure 13:**
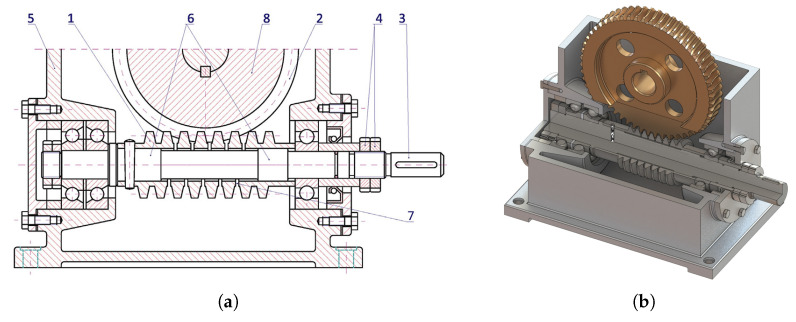
Worm gear drive with locally axially adaptive worm. (**a**) Worm gear drive structural design. (**b**) Worm gear drive model.

Another design solution in which a worm has been modified is a zero-backlash worm manufactured according to patent P.137 131 [28]. A characteristic feature for this type of drive is that the worm has a hollow axial hole with diameter equal to, or slightly larger than the diameter of the cut-outs. Most significantly, however, the bottom of the worm thread coil is cut along the helical line, in the centre of the worm itself (Figure 13).

As a result of this operation, the worm becomes a particular kind of spring with high axial stiffness and anchored ends. As a consequence, the component is locally axially adaptive and it is possible to change the pitch of the worm by compression or tension. This type of worm gear drive has many advantages, including simplicity of design, along with its uncomplicated mechanism of backlash adjustment, which enables a change of setting even while the drive is in operation. The mating components of the drive are well lubricated in the meshing zone regardless of the direction of rotation. The axial susceptibility of the worm favourably influences the reduction of dynamic surplus, as well as damping vibration.

**Figure 14:**
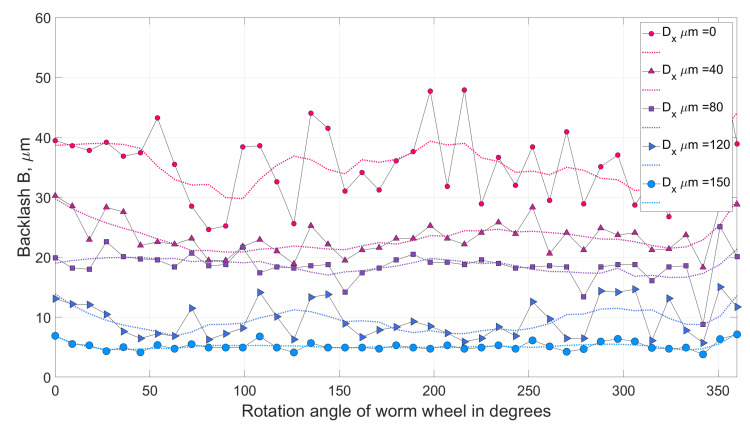
Backlash *B* adjustment results for WG1 worm gear drive for different values of adjustment settings $D_{x}$∈ {0, 40, 80, 120, 150} μm.

Many issues concerning the design of worm gear drives with backlash adjustment have been both solved and described by Kacalak et al. in studies [16,19,26,27]. In study [29], the influence of backlash adjustment on reducing its statistical values and characteristics (Figure 14) was determined for a drive with a worm seated on a mandrel, and axially adaptive in its central section (a worm gear drive marked with the symbol **WG1**). Although the results were considered good, relatively high worm susceptibility limits excessive load capacity, due to the fact that deformation of the active part of the worm coil occurs during recurrent operations, which deteriorates positioning accuracy.

Combining an axially flexible worm, but one possessing higher axial stiffness, with a worm wheel with a displaceable or deformable rim, can contribute a beneficial reduction of backlash and reduce the impact of worm susceptibility on positioning deviations.

**Figure 15:**
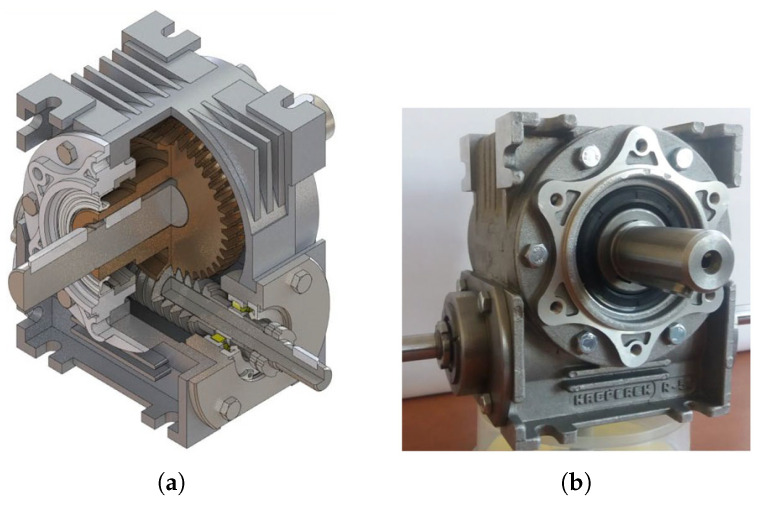
Worm gear drive with locally axially adaptive worm as experimental subject. (**a**) Worm gear drive model. (**b**) WG2 worm gear drive used in experimental research.

Experiments were conducted in which the experimental subject was another worm gear drive with a locally axially susceptible worm, designated **WG2** (Figure 15).

**Figure 16:**
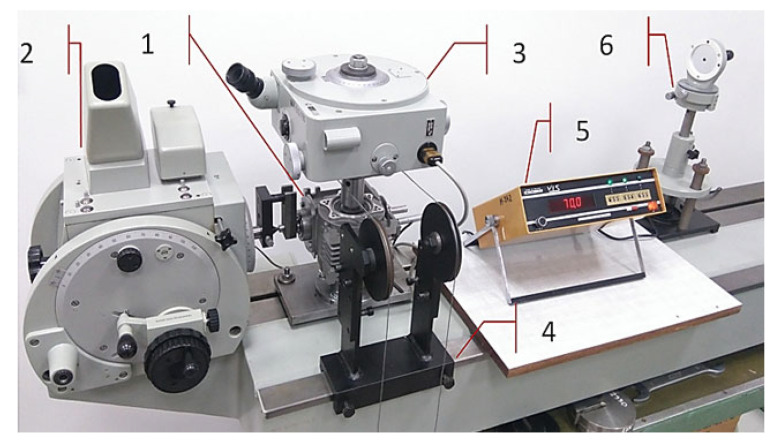
Testing stand—general view: 1—worm, 2—optical measuring head P3, 3—autocollimator, 4—worm wheel loading mechanism, 5—induction sensor with displacement gauge, 6—autocollimation mirror.

Research on the influence of regulation settings on the backlash value was carried out on an experimental stand (Figure 16) for two values of worm axial compression, which assumed, respectively, a value equal to the maximum value of compression (resulting from reaching the state when the lowest level of backlash in the set of all values during the movement of the drive reaches the a value of zero), and the value decreased by 90 μm of the settings from the value assumed to be the maximum.

The values of the parameters characterising the worm were as follows: worm with a conical helical surface ground by a disc grinding wheel with a rectilinear outline of the active surface in the axial section; outline angle α = 20${}^{\circ }$, coiling to the right, pitch diameter $d_{1}$ = 24 mm, diameter of coil vertices $dw_{1}$ = 28 mm, core diameter $dr_{1}$ = 19. 2 mm, coil helix angle γ = 4${}^{\circ }$46${}^{\prime }$, diameter ratio *q* = $d_{1}$/*m* = 12, pitch *s* = πm = 6.283 mm, axial pitch $p_{x}$ = 6.28 mm, normal pitch $p_{n}$ = 6.258 mm, axial module $m_{o}$ = 2 mm, normal module $m_{n}$ = 1.993 mm. The values of the basic parameters characterising the worm wheels used for experimental verification of the new solutions were as follows: number of teeth $z_{2}$ =38, pitch diameter $d_{2}$ = 76 mm, vertex diameter $dw_{2}$ = 80 mm, diameter of cut-out $dr_{2}$ = 71.2 mm, rim width *b* = 22 mm.

The state of meshing in a worm gear drive is variable during one rotation of the worm wheel, while the local adaptability of deformable zones of drive components in the meshing zone is limited regarding various solutions for minimising backlash.

The main scientific problem here is to determine the principles of achieving a compromise between the efficiency of backlash reduction and the required load capacity of worm gear drives, as well as the effects of local deformations of adaptive zones being impacted by an external load.

Adaptive or regulatory reduction of backlash in a meshing zone does not increase the resistance to motion until a state is achieved when the lowest backlash value for a specific tooth combination still remains positive or reaches zero.

**Figure 17:**
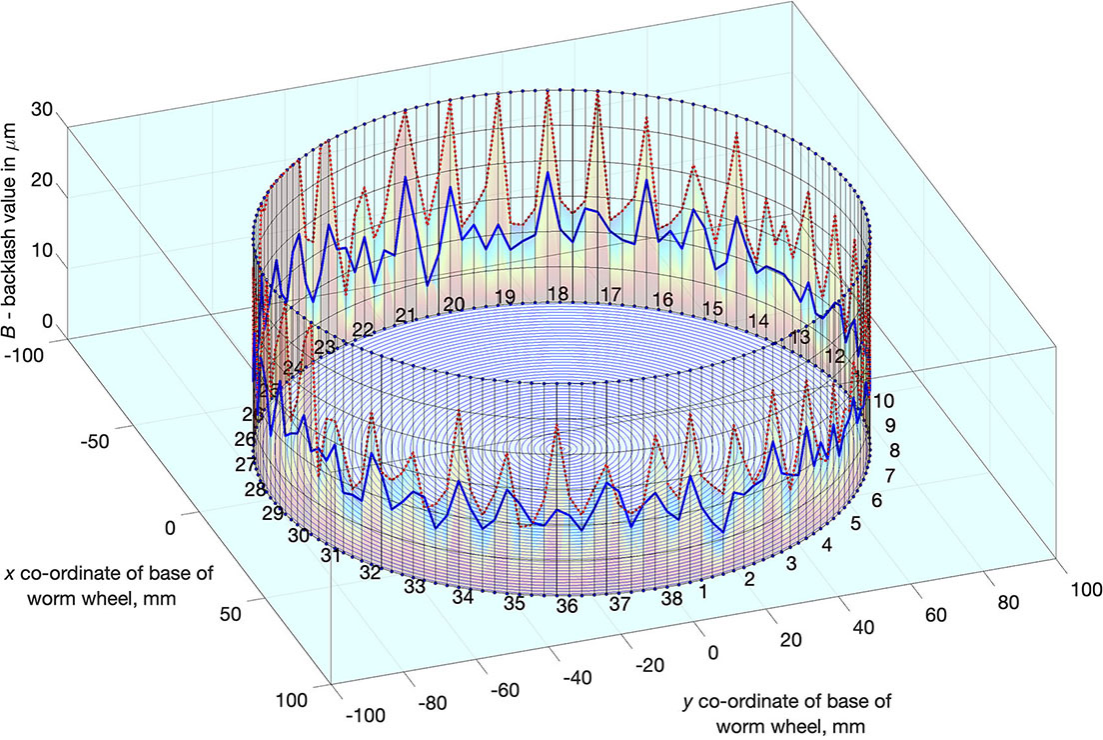
Backlash for positions of successive worm wheel teeth in the centre of the meshing zone; the red line represents backlash values before adjustment; the blue line—after adjustment.

**Figure 18:**
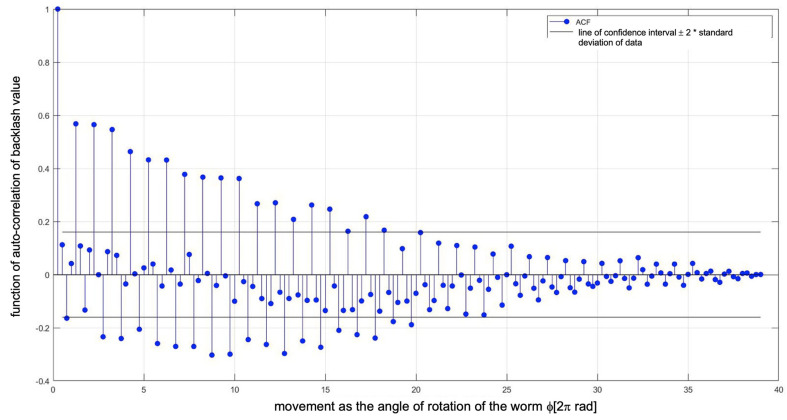
Length of autocorrelation of backlash values.

From the backlash analysis (Figure 17), it can be seen that an increase in the axial compression of the worm, i.e., the adjustment setting, causes a significant reduction in the backlash value. A low standard deviation value is important here as it means a more effective minimisation of backlash. The length of the autocorrelation of the backlash value (Figure 18), reaching even a value corresponding to half of the worm wheel rotation (19 turns of the worm), indicates compound inaccuracies resulting from the radial runout in the worm wheel machining process (under the technological conditions of the manufacturer of precision worm gear drives).

Another objective was to determine the relationship between drive parameters and axial deformation of the worm during loading of the drive (axially adaptive worm) and motion resistance. It was taken into consideration that:

- with an increase of the setting parameter, namely axial compression of the worm, drive backlash decreases,
- as worm stiffness in the axial direction increases, backlash also increases.

**Figure 19:**
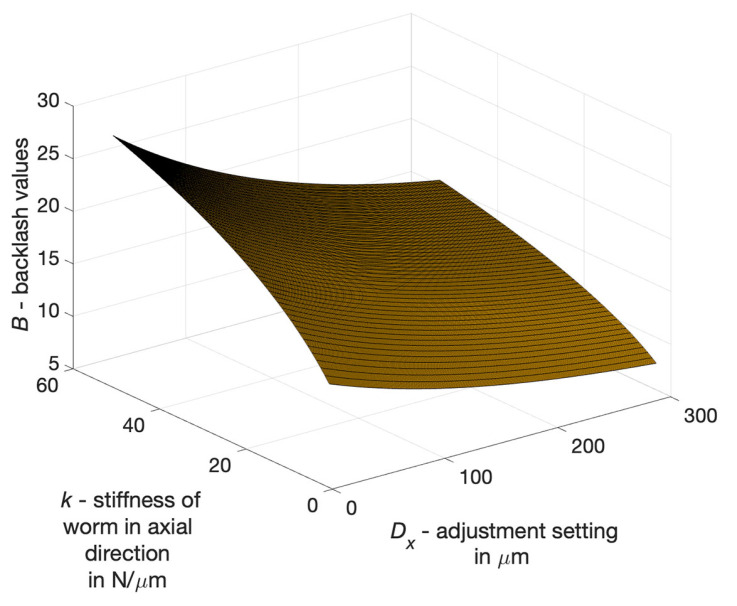
Backlash values *B* of worm gear drive as a function of the adjustment setting $D_{x}$ and worm stiffness *k* in the axial direction.

The backlash value *B* depends on the axial deformation of the worm $f_{x}$ being subjected the influence of a load and motion resistance *M*, as well as the setting $D_{x}$ that minimises backlash. Axial deformation of the worm depends on its axial stiffness and load, along with motion resistance (Figure 19) and is expressed by (6):

(6) $$\begin{array}{c} B=c_{1}\cdot \left(1-e^{-a\cdot k^{0.5}}\right)\cdot \left(7+10\cdot e^{-b\cdot D_{x}}\right)\left[\mu m\right] \end{array}$$

where:

- *k*—axial stiffness expressed in N/μm, depending on the module *m*, pitch diameter *d* and the axial length of the helical intersection,
- $D_{x}$—the setting representing the axial compression of the worm, expressed in μm,
- $c_{1}$ = 3.05, *a* = 0.11 and *b* = 0.005—constants in the equation B=f(k,n).

Axial compression of the worm is carried out only until the smallest backlash value for one of the worm wheel angular positions reaches zero. In this state, an external compressive force does not change forces in the meshing zone resulting from gear drive loading. Gear drives of this type are designed for positioning applications where the external load is relatively small. In other applications, when the load is quite high, local deflections of the coil favourably equalise the loads in the meshing zone.

**Figure 20:**
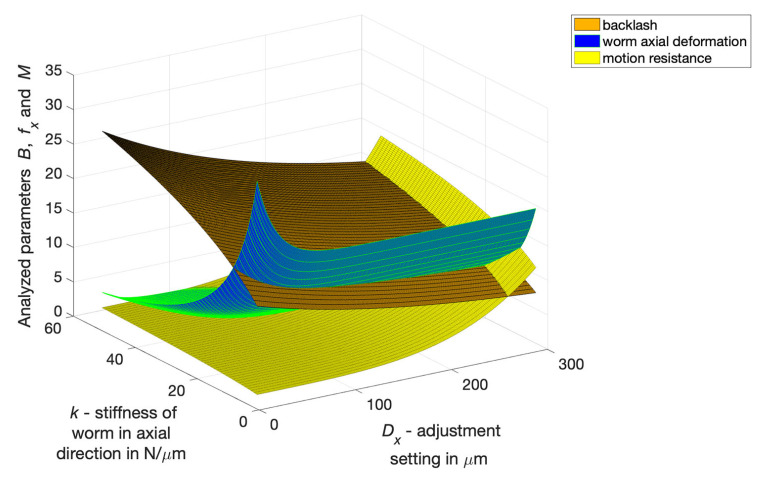
Backlash *B* in μm as a function of adjustment setting *n* and axial stiffness *k* of the worm without a load (brown area), axial deformation $f_{x}$ of the worm for the tested drive (green area) and motion resistance *M* in Nm.

The axial deformation of the worm $f_{x}$ depends on its axial stiffness, load and motion resistance. The relationship for the worm $f_{x}$ in the axial direction with a load (Figure 20) is expressed by Equation (7):

(7) $$f_{x}=\frac{c_{2}}{k}\cdot \left(1+e^{-d\cdot D_{x}}\right)\left[\mu m\right]$$

where: $c_{2}$ = 205 and *d* = 0.05 are constants in the equation B=f(k,n) and depend on the load.

**Figure 21:**
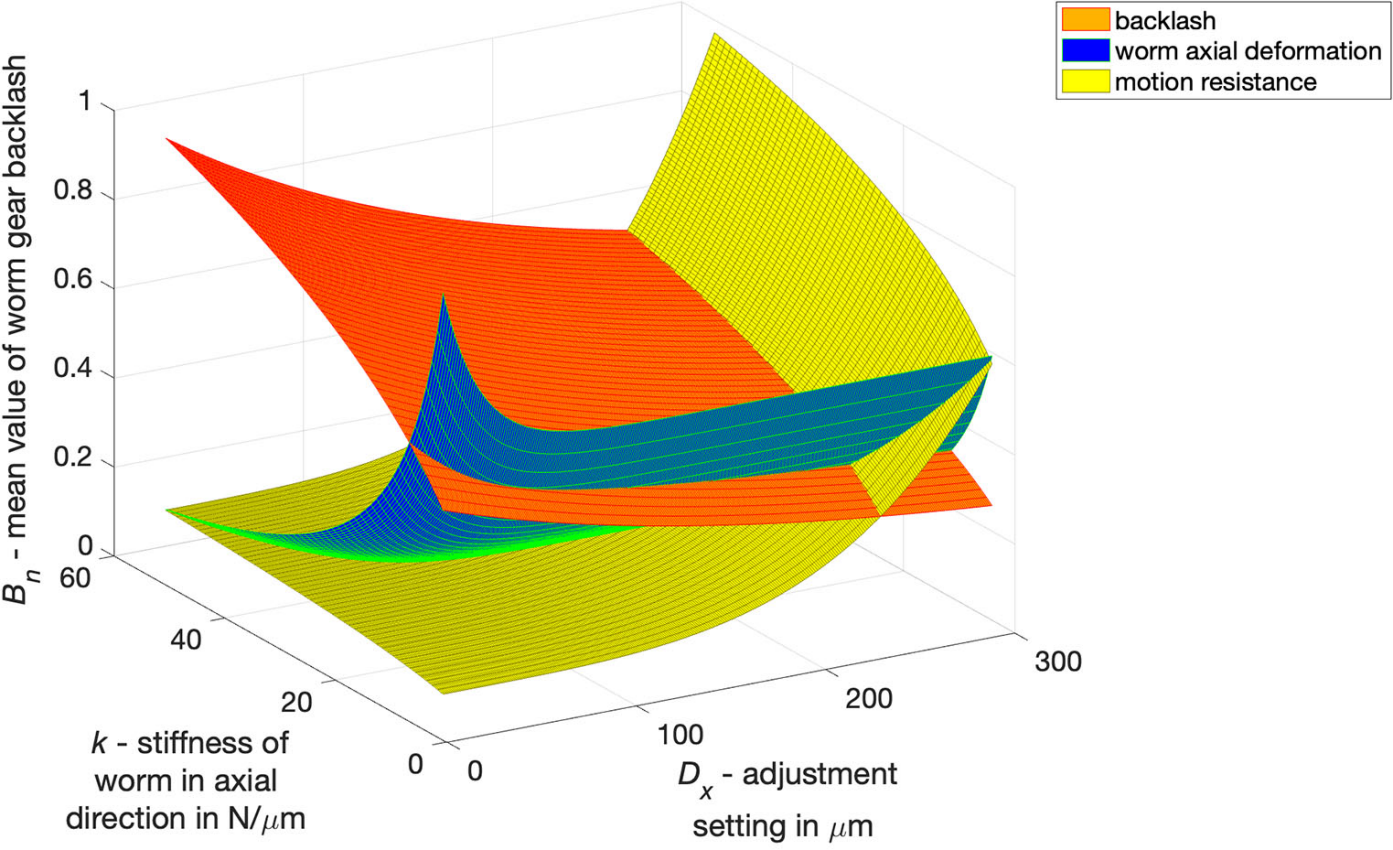
Influence of axial stiffness *k* and adjustment setting $D_{x}$ on backlash *B*, worm axial deformation $f_{x}$ and drive motion resistance *M* (after normalising *B*, $f_{x}$ and *M* to the interval <0, 1>).

The relationship describing the influence of axial stiffness *k* and setting *n* causing a decrease of worm axial pitch (backlash minimisation) on drive motion resistance (moment of motion resistance *M*) (Figure 21) is determined as follows in Equation (8):

(8) $$M=c_{3}\left(1-e^{-w\cdot k^{0.5}}\right)\cdot {\left(1+\frac{D_{x}}{D_{x_{lim}}}\right)}^{4}\left[Nm\right]$$

where: $c_{3}$ = 4.05, *w* = 0.21 and $D_{x_{lim}}$ = 200 μm—constants in the equation M=f(k,n).

It should be stressed that reduction of backlash should only be carried out up to a point where, in a certain reciprocal position of the worm and worm wheel, the smallest backlash value reaches zero. Further axial compression of the worm is not justified, since it causes, in certain angular positions, an increase in motion resistance due to the necessary deformation of the worm coil. Therefore, decreasing backlash up to this limit value does not significantly increase motion resistance.

Lubrication of the gear drive influences motion resistance and it is a characteristic that depends on lubrication conditions in the meshing zone. Although the value of resistance decreases, the character of the relationship remains similar. Cutting the bottom of the worm screw coils favourably improves the flow of lubricating oil in the meshing zone.

A multi-criteria selection of geometric parameters of a worm gear drive was carried out(module, length of helical line of coil bottom section, and width of this section) using as criteria the minimisation of backlash values min(B), the minimisation of worm axial deformation $min(f_{x})$, and the minimisation of drive motion resistance min(M).

The normalised values of *B* were determined from Equation (9). The normalised values of $f_{x}$ and *R* were calculated in a similar manner.

(9) $$B_{n}=\frac{B-B_{min}}{B_{max}-B_{min}}$$

The arithmetic mean and geometric mean of the normalised values of the partial criteria were then determined.

**Figure 22:**
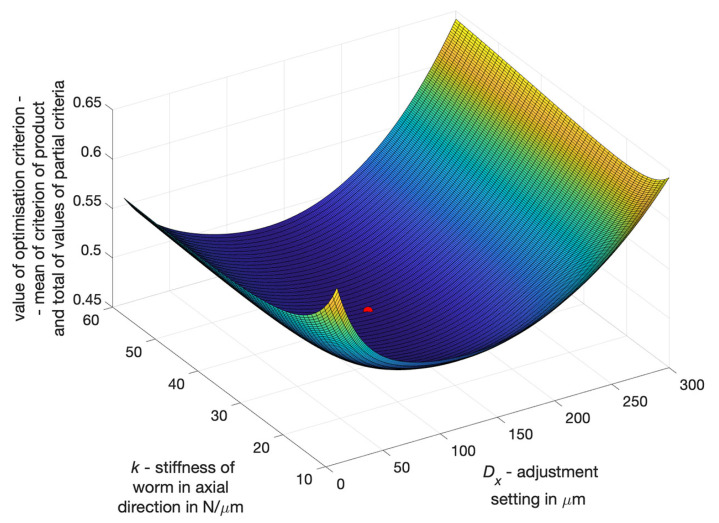
Result of multi-criteria selection of worm axial stiffness and adjustment setting for a specific worm gear drive.

The result of optimising the axial stiffness of the worm and the adjustment setting (Figure 22) was determined (10) as the minimum of the product of the arithmetic mean value $S_{A}$($B_{n}$, $f_{x_{n}}$, $M_{n}$) of the sum of the normalised values of all partial criteria ($B_{n}$, $f_{x_{n}}$, $M_{n}$) and the geometric mean value $S_{G}$($B_{n}$, $f_{x_{n}}$, $M_{n}$) of the partial criteria ($B_{n}$, $f_{x_{n}}$, $M_{n}$).

(10) $${\left(D_{x},k\right)}_{opt}=min\left(S_{A}\cdot S_{G}\right)$$

where: $S_{A}$ = ($B_{n}+f_{x_{n}}+M_{n}$)/3, $S_{G}$ = ($B_{n}\cdot f_{x_{n}}\cdot M_{n}$)${}^{\left(1/3\right)}$, $B_{n}$—normalised backlash value for a given worm gear drive characteristics and the adjustment setting, $f_{x_{n}}$—the normalised value of axial deformation of the worm for a given load and setting of the drive, $M_{n}$—normalised value of motion resistance of the drive for specific parameters of the drive and the adjustment setting and load of the drive.

The following conclusions can be drawn from the conducted analyses:

- The lowest backlash value can be obtained at the maximum setting and the lowest permissible level of axial stiffness of an adaptive worm with the cut of the coil bottoms in the middle zone situated on the mandrel;
- The axial deformation of the worm decreases as the setting increases;
- The motion resistance of the drive depends mainly on the adjustment setting (and on the worm stiffness to a much lesser extent), with the increase of the setting increasing the motion resistance, whereby the motion resistance undergoes a significant increase for the adjustment settings, greater than those required to achieve zero backlash in one position relative to the worm and worm wheel).

## 4. Conclusions

The article presents the development of the basis of construction and optimisation of worm gears enabling minimisation of backlash in the meshing zone and increasing kinematic accuracy. As a result of the conducted experiments, it was concluded that the selection of features and design parameters of drive assemblies should depend on the result of polyoptimisation. In this task, a compromise is expected between the minimisation of backlash, the minimisation of axial deformation in the meshing zone, and the minimisation of resistance to drive movement. The result of the optimisation procedure depends on the axial susceptibility of the worm and the adjustment setting.

- In a worm gear drive with a locally axially adaptive worm and a worm wheel with a deformable rim, significant reduction of backlash can be achieved, which in high precision drives (with backlash < 30 micrometers) (Figure 17) enables more than a two-fold reduction of the average backlash value and more than a three-fold decrease of the standard deviation of local backlash values. For low-precision drives, a high degree of backlash reduction can be easily achieved. A more difficult task, however, is to obtain a high degree of backlash reduction in high precision drives.
- Selection of worm gear drive design features and parameters should depend on the result of polyoptimisation, which consists of determining features depending simultaneously on expectations concerning the following: the minimum backlash values, the minimum values of axial deformation and the minimum motion resistance to movement of the drive, all of which depend on the worm gear drive’s geometrical parameters and the adjustment setting entered (Figure 22).
- Local radial deformations of the worm wheel rim do not deteriorate axial susceptibility of the worm gear drive and have a positive impact on kinematic accuracy. At the same time, they increase the axial stiffness of the worm with a locally cut coil bottom, with the possibility of filling this cut-out with an adaptive material. This will allow one to obtain sufficient load capacity of the worm gear drive.

## Data Availability

Data sharing is not applicable to this article.
